# Supplementary material for: In Vivo PET Detection of Lung Micrometastasis in Mice by Targeting Endothelial VCAM-1 Using a Dual-Contrast PET/MRI Probe
Source: Int J Mol Sci. 2024 Jun 28;25(13):7160. doi: 10.3390/ijms25137160 (PMC11241628; doi:10.3390/ijms25137160)
Supplement: Supplementary file 1 [file ijms-25-07160-s001.zip › ijms-3056414-supplementary.pdf]

International Journal of *Molecular Sciences*

MDPI

Article

In Vivo PET Detection of Lung Micrometastasis in Mice by Targeting Endothelial VCAM-1 Using a Dual-Contrast PET/MRI Probe

Supplementary Material

S1: Specific binding of VCAM-MPIO and mean diameter of metastasis

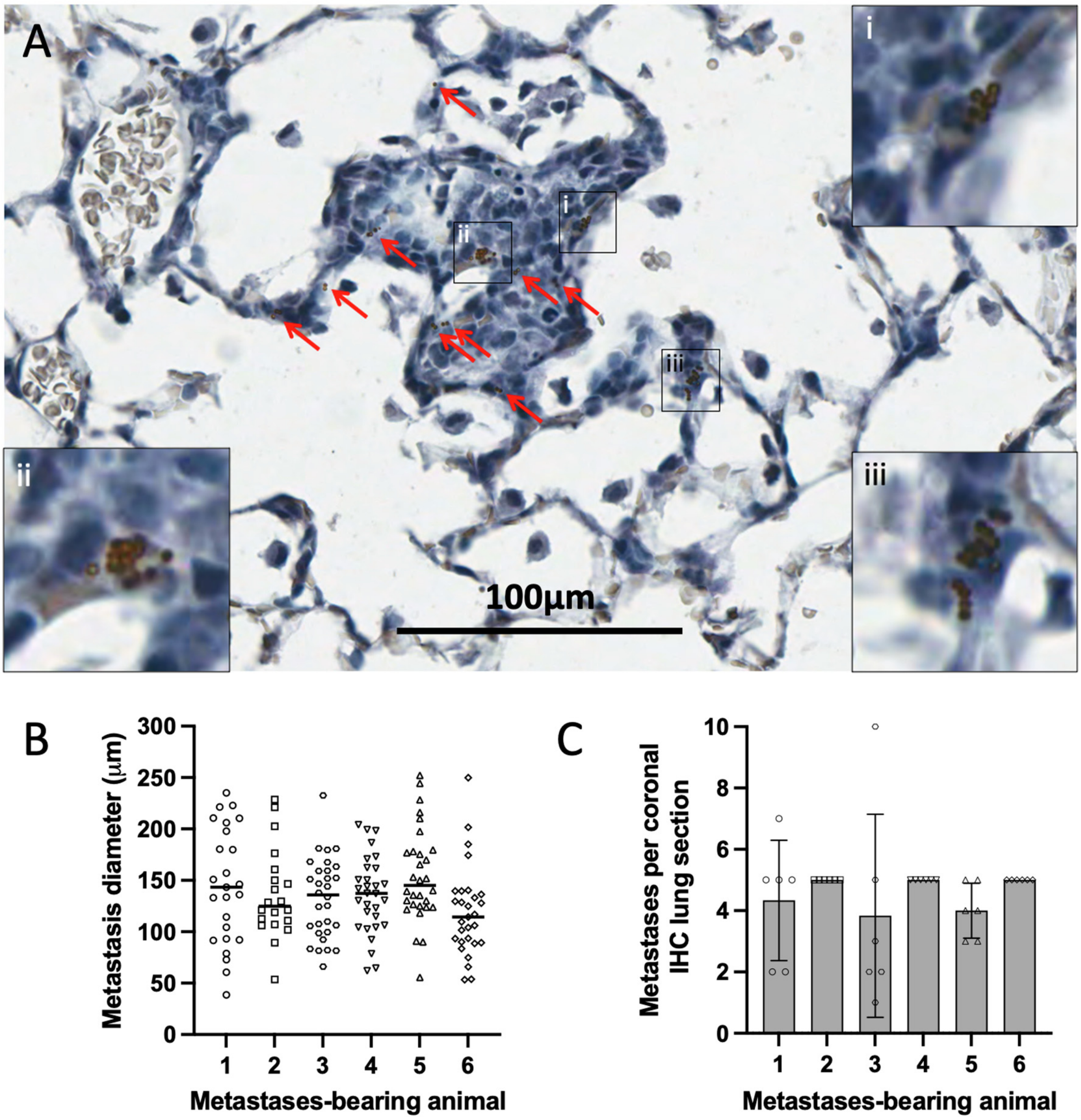

Figure S1.

(**A**) Representative image of lung tissue acquired from the metastatic mouse model (day 10) 10 min post-intravenous injection of VCAM-MPIO. The image exclusively features nuclear counterstaining (in blue) to enhance the

differentiation of MPIO particles. A substantial number of MPIO are associated with the metastasis microvasculature (red arrows and higher magnification areas). (**B**) Column chart showing the area-derived diameters of metastases (137.01 ± 45.17 μm) scored manually from 6 pulmonary metastases-bearing animals at day 10, obtained from 6 evenly spaced (1 mm apart) coronal parallel sections per mouse. (**C**) Column bar chart showing the count of metastases scored from each immunohistochemical section in individual mice, corresponding to the data presented in (**B**).

S2: *In vivo* PET imaging

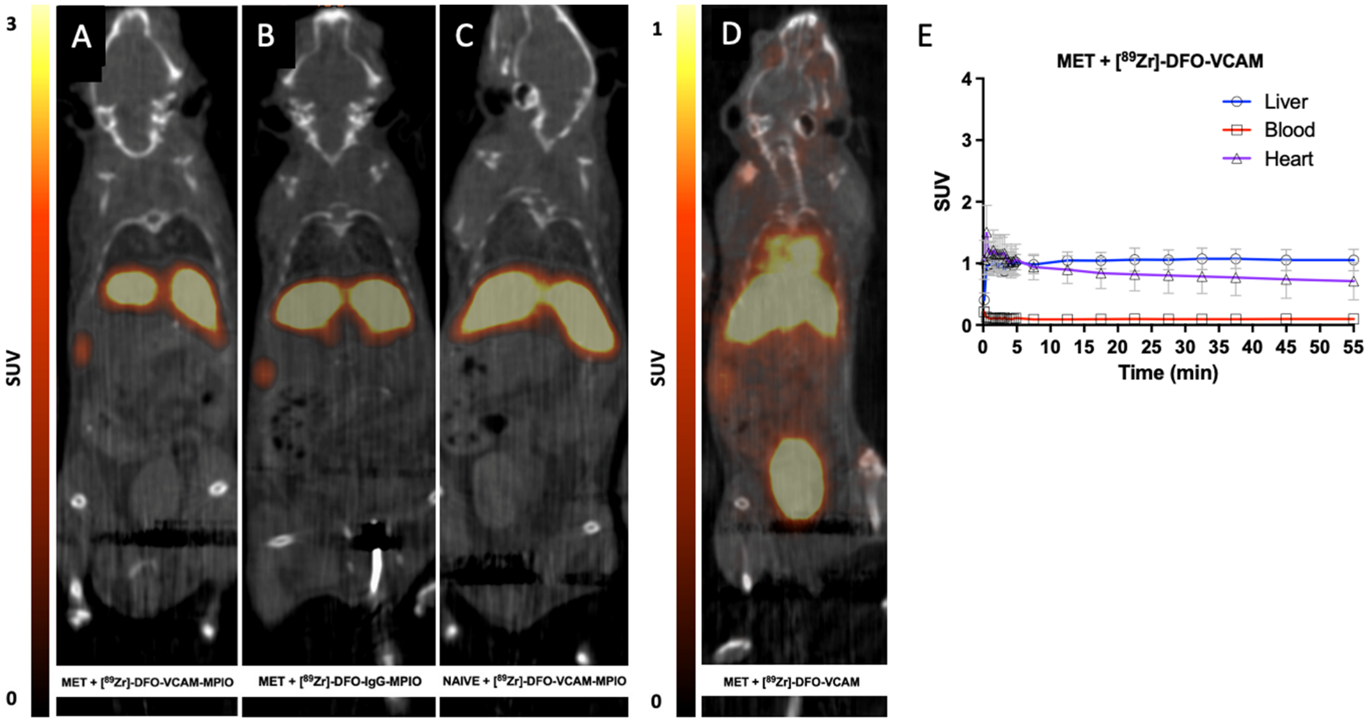

Figure S2

(A) Single plane, static, non-gated PET image at 55 min from a mouse bearing pulmonary metastases and injected intravenously with [89Zr]-DFO-VCAM-MPIO, as shown in Figure 3A. No visible signal is evident in the lungs owing to very strong signal from the liver and spleen, which prevents the use of a colour scale for the *in vivo* image that would allow visualization of the lung signal owing to partial volume effects. (**B**) Single plane, static, non-gated PET image at 55 min from a mouse bearing pulmonary metastases and injected with the isotype control agent [89Zr]-DFO-IgG-MPIO, as shown in Figure 3B. Strong uptake of radioactivity is again apparent in the liver and to a lesser extent at the spleen. **(C)** Single plane, static, non-gated PET image at 55 min from a naive (control) mouse injected with [89Zr]-DFO-VCAM-MPIO showing strong uptake of radioactivity in the liver and to a lesser extent in the spleen. **(D)** Single plane, static, non-gated PET image at 55 min from a metastasis bearing mouse injected with [89Zr]-DFO-VCAM (i.e. non-MPIO conjugated antibody) showing high levels of radioactivity in the heart, liver, bladder and also to a lesser extent in the spleen and bones. (**E**) Prolonged circulation of [89Zr]-DFO-VCAM (i.e. non-MPIO conjugated antibody) in the blood, and consequently high levels of radioactivity in the heart, was observed throughout the first hour after administration in all mice in the group.

Although the single plane static PET images do not appear to show any [89Zr]-DFO-VCAM-MPIO specific signal in the lungs of metastasis-bearing mice (Figure S3A), when all of the data from the

3D PET acquisition are combined (i.e. true sum of all of the planes of the PET data across the whole lung) radioactivity within the lungs can clearly be visualised (Figure 3A). Thus, on the single plane images the signal from the lungs is extremely weak compared to the strong signal from the liver, and this precludes it's visualisation. Nevertheless, we have experimentally measured significantly higher radioactivity uptake in the lungs of mice with pulmonary metastases injected with [^{89}Zr]-DFO-VCAM-MPIO compared to all other groups (Figure 3E-G) and this can be visualised using the maximum intensity plots (Figure 3A-B). Naïve (control) mice injected with [^{89}Zr]-DFO-VCAM-MPIO also showed uptake primarily in the liver and spleen (Figure S3C). Metastasis-bearing animals injected with [^{89}Zr]-DFO-VCAM (i.e. non-MPIO conjugated antibody) showed extensive circulation of the agent in the blood for the first hour (Figure S3D-E), and high levels of radioactivity in the heart. Liver and bladder also showed high uptake of radioactivity in these animals, whilst lower levels of radioactivity were evident in the spleen and the bones (Figure S3D).

S3: Assessment of MPIO circulatory half-life

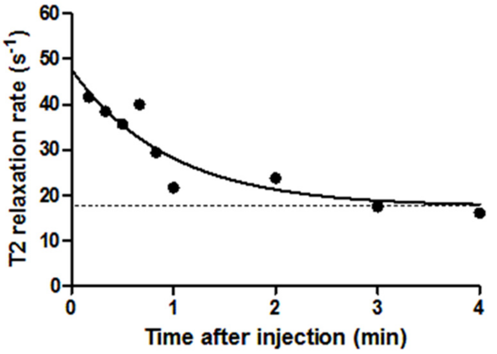

Figure S3

Graph showing T_2 relaxivity values of blood samples at different time points after administration of 1.08 μm MPIO, at a dose of 4 mg Fe/kg body weight. The clearance half-life was calculated to be 39.9s (K = 0.017 s^{-1}, Y0 = 47.80 Hz). Curve is fitted as a single phase exponential decay constrained to a plateau at the average naïve blood sample values (baseline; dashed lines).

To assess the MPIO circulatory half-life, 7-9 week old female CD1 mice (n = 9; Charles River, UK) were anesthetised with 2-3% isoflurane in O_2 and injected intravenously via a tail vein with BSA-MPIO at a constant dose of 4 mg of iron per kg of body weight in 100 μL sterile PBS. At different time points after administration of BSA-MPIO, mice underwent a thoracotomy, followed by cutting of the inferior vena cava and aorta below the lungs to allow the blood to pool inside the thoracic cavity. Blood was collected with a syringe containing 5 μL of heparin sodium (25,000 I.U./mL, Wockhardt, Wrexham, UK). For early time points, surgery was performed prior to injection of particles, leaving the diaphragm intact. Blood samples were obtained at 10, 20, 30, 40, 50, 60, 120, 180 and 240 s after MPIO injection (n = 1 per time point). Subsequently, the blood samples were solidified and relaxivity assessed by MRI in order to determine the circulatory half-life (see below) of MPIO in the blood. Blood samples from naïve mice were used to assess relaxivity of normal blood (n = 5).

Blood samples were oxygenated by bubbling O_2 gas through them for 5 min, in a sealed tube on a rolling platform. Subsequently, blood samples were mixed with 1% agarose solution in PBS (1:1 ratio) at 40.5°C to a final volume of 300 µL. Samples were allowed to set in microcentrifuge tubes and stored at 4°C. T_2 relaxivity measurements were made within 24 h using a 4.7 T horizontal bore magnet (Agilent Technologies, Santa Clara, CA, USA) with a 25 mm quadrature birdcage coil (Rapid Biomedical, Wurzburgy, Germany); an array of 100 µs hard 90° and 180° pulses were used with a TR = 1000 ms, 2000 complex points and 8 averages. Circulatory half-life was determined from the fitted one phase decay curve, constrained to a plateau at the average naive blood sample value (baseline; Figure S1a). The half-life of MPIO in the circulation was estimated to be 39.9 s (K = 0.017 s^{-1}, Y_0 = 47.8 Hz).

S4: Assessment of autoradiograph and immunohistochemical image for colocalization of foci with [^{89}Zr]-DFO-VCAM-MPIO and micrometastases.

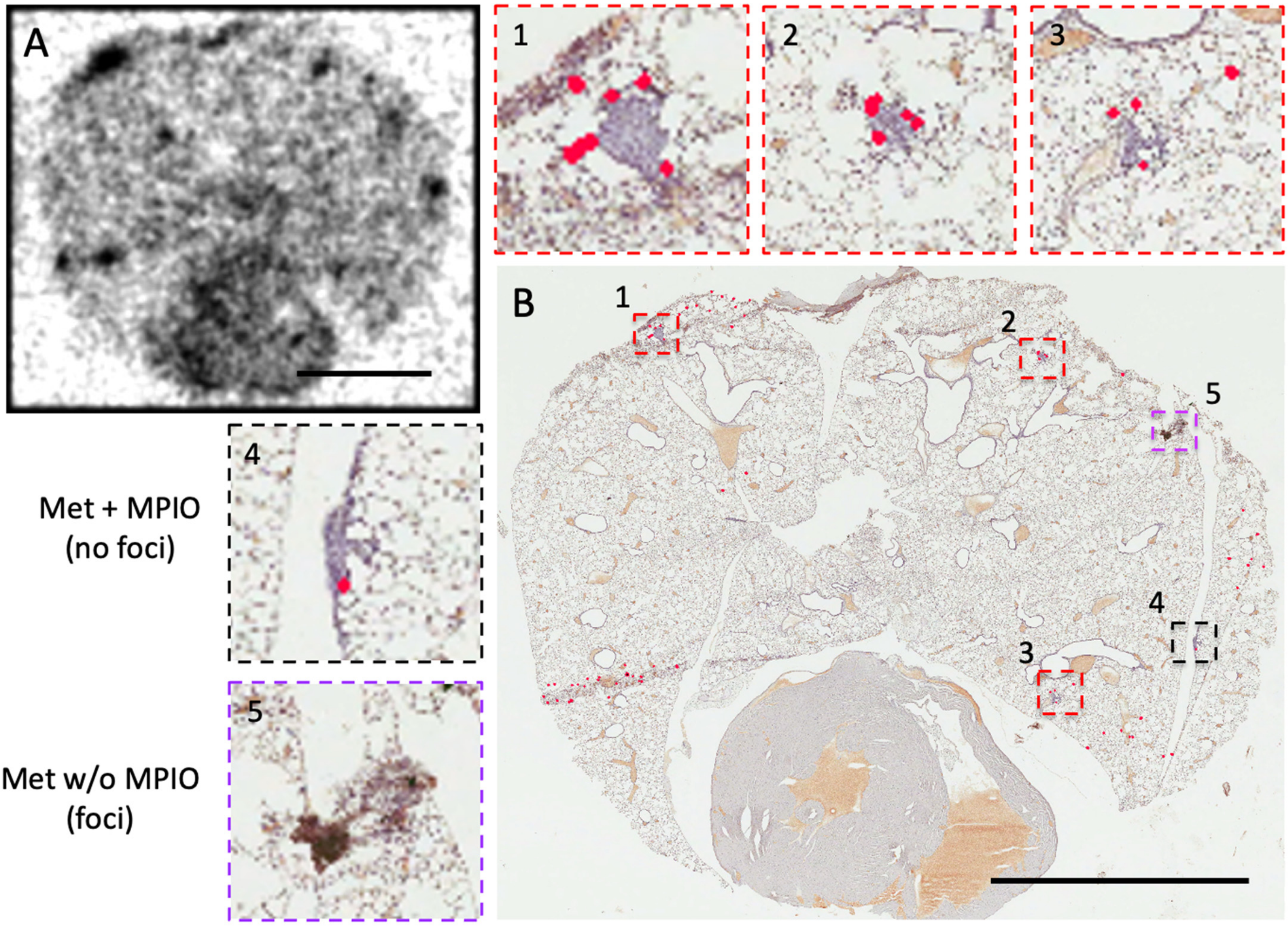

Figure S4

(**A**) Autoradiographic image from lung tissue section after 24 h exposure and (**B**) immunohistochemical image from the same lung section manual inspected under magnification. (**B1-3**) Foci of high activity on the autoradiographic plate co-localise with VCAM-1 expressing micrometastatic areas that associated with manual scored MPIO (red dashed squares;

small red marks on tissue). (**B4**) Metastasis with low score of MPIO that did not show high activity on the autoradiographic plate (black dashed square; small red mark on tissue). (**B5**) Metastasis scored with 0 presence of MPIO that correlated with moderate activity foci (purple dashed square). Some areas with high activity foci in the autoradiographic image (**A**) did not correlate with apparent metastases (**B**), but did correlate with areas where MPIO were observed (small red dots on tissue in (**B**)). Scale bars = 5 mm.

S5: Assessment of brain metastasis detection at lower dose [^{89}Zr]-DFO-VCAM-MPIO

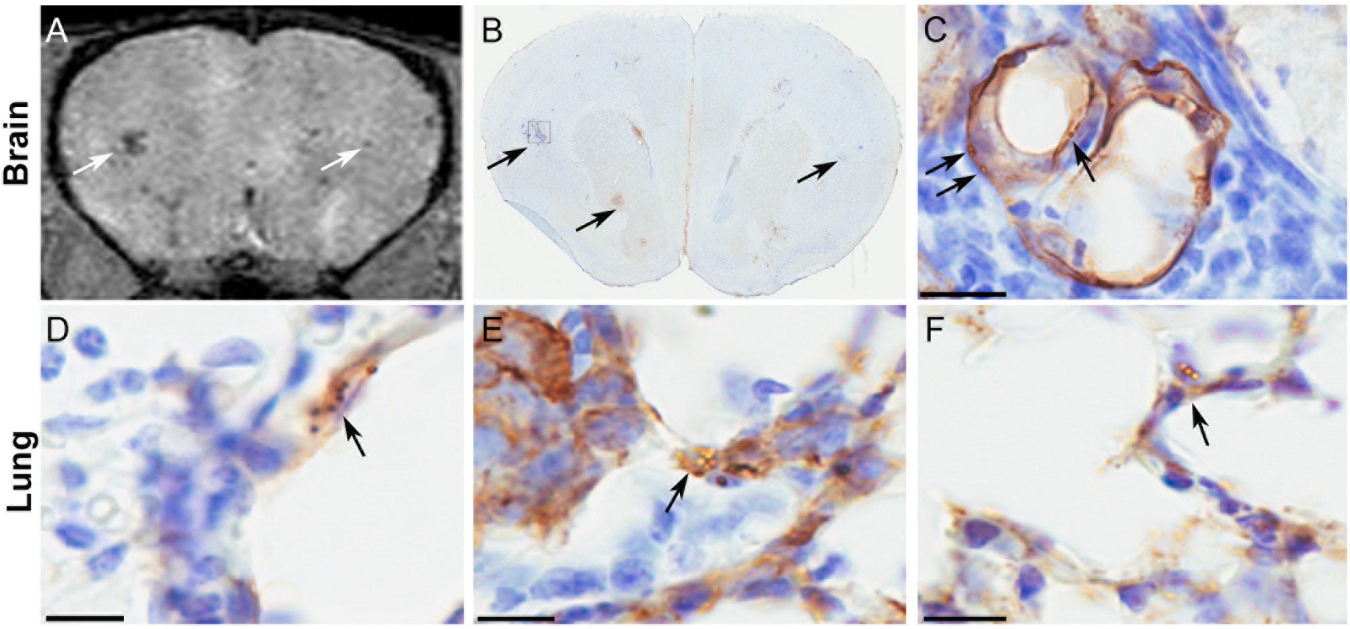

Figure S5

(**A-C**) Co-localization of [^{89}Zr]-DFO-VCAM-MPIO and brain metastases in 4T1-GFP model at low-dose (1.6mg Fe/kg body weight). Co-localization of focal hypointensities on a T_2*-weighted multi-gradient echo image (**A**; arrows) with histological detection of brain micrometastases (**B**; arrows); (**C**) zoomed picture of the inset shown in **B**, arrows indicate MPIO on VCAM-1 positive (brown) vessel associated with micrometastasis. Scale bar = 20 µm. (**D-F)** Lung sections showing presence of [^{89}Zr]-DFO-VCAM-MPIO (arrows) within VCAM-1-positive vessels (brown stain) closely associated with lung micrometastases. Scale bar = 10 µm.

To determine whether the lower dose of targeted MPIO optimised for lung detection of metastases is sufficient to enable detection of brain metastases and in the presence of an additional metastatic load in the lungs, a model of dual (brain and lung) metastases was developed. Female BALB/c mice (7-10 weeks old, n = 4; Charles River, UK) were anaesthetized with isoflurane (2.0-3.0%) in oxygen. Mice were injected intracardially with 5×10^4 4T1-GFP into the left ventricle under ultrasound guidance (Vevo 3100, FUJIFILM VisualSonics, Toronto, ON, CA), as described previously (Serres *et al.*, 2012 *PNAS.* 109: 6674–9), and also with 5×10^4 4T1-GFP cells intravenously via a tail vein, as described in this paper.

At day 14 after tumour cell injections, mice were anaesthetized with 2–3% isoflurane in 70% N_2O:30% O_2 and injected intravenously via a tail vein with [^{89}Zr]-DFO-VCAM-MPIO (1.6 mg Fe/kg in 100 µL sterile PBS). MRI data from the brain were acquired using a 9.4 T horizontal, wide-bore superconductive MRI system (Agilent Technologies Inc., Santa Clara, CA, USA). Approximately 1 h after contrast agent administration, T_2*-weighted multi-gradient echo 3D (MGE3D) data were acquired to detect bound MPIO in the brain. MGE3D sequence parameters were as follows: excitation angle = 15°; repetition time (TR) = 65.1 ms; time of the 1st echo (TE1) = 2.5 ms; echo separation time (TE2) = 4 ms (even echoes acquired in the reverse k-space direction, during opposite read gradient); imaging bandwidth (SW) = 100 kHz (acquisition time = 2.56 ms); number of echoes (NE) = 15. As a control, naïve mice were injected with [^{89}Zr]-DFO-VCAM-MPIO (n=2), as above. Following MRI, mice were perfusion-fixed, as described previously (Serres, *et al.* 2012 *PNAS.* 109: 6674–9), and brains taken for immunohistochemistry. Sections were cut and stained for immunohistochemical detection of VCAM-1 and co-localisation with [^{89}Zr]-DFO-VCAM-MPIO, as described by Serres *et al.*, 2012.

In all mice injected with tumour cells, focal hypointensities were seen within the brains, indicating binding of [^{89}Zr]-DFO-VCAM-MPIO, in accord with previous findings using VCAM-MPIO at the higher dose of 4mg Fe/kg body weight (Serres, *et al.* 2012 *PNAS.* 109: 6674–9). On histological assessment, co-localisation of the MRI-detectable hypointensities with micrometastases was evident (Figure S5A-B) and MPIO were observed within VCAM-1 positive vessels (Figure S5C). Subsequent histological assessment of lung tissue from the same animals, confirmed the presence of micrometastases and binding of [^{89}Zr]-DFO-VCAM-MPIO to VCAM-1-positive vessels (Figure S5D-F).

These data demonstrate efficacy of the lower dose of VCAM-1 targeted MPIO used in this study for detection of brain metastases and, further, that the additional presence of lung metastases does not preclude sensitive detection of brain metastases.
